# Defining constipation to estimate its prevalence in the community: results from a national survey

**DOI:** 10.1186/s12876-019-0994-0

**Published:** 2019-05-21

**Authors:** Barry L. Werth, Kylie A. Williams, Murray J. Fisher, Lisa G. Pont

**Affiliations:** 1 Sydney Nursing School, University of Sydney, Sydney, NSW 2006 Australia; 2 Discipline of Pharmacy, Graduate School of Health, University of Technology Sydney, Sydney, NSW 2007 Australia

**Keywords:** Prevalence, Measurement, Adults, Constipation, Epidemiology

## Abstract

**Background:**

Different definitions of constipation have been used to estimate its prevalence in the community but this creates difficulties when comparing results from various studies. This study explores the impact of different definitions on prevalence estimates in the same population and compares the performance of simple definitions with the Rome III criteria.

**Methods:**

The prevalence of constipation in a large nationally representative sample of community-dwelling adults was estimated using five simple definitions of constipation and compared with definitions based on the Rome III criteria. The sensitivity, specificity, and positive and negative predictive values, were calculated for each definition using the Rome III criteria as the gold standards for chronic and sub-chronic constipation.

**Results:**

Prevalence estimates for the five simple definitions ranged from 9.4 to 58.9%, while the prevalence estimates using the Rome III criteria were 24.0% (95%CI: 22.1, 25.9) for chronic constipation and 39.6% (95%CI: 37.5, 41.7) for sub-chronic constipation. None of the simple definitions were adequate compared to the Rome III criteria. Self-reported constipation over the past 12 months had the highest sensitivity (91.1%, 95%CI: 88.8, 93.4) and negative predictive value (94.5%, 95%CI: 93.1, 96.1) compared to the Rome III criteria for chronic constipation but an unacceptably low specificity (51.3%, 95%CI: 48.8, 53.8) and positive predictive value (37.1%, 95%CI: 34.4, 39.9).

**Conclusions:**

The definition used to identify constipation within a population has a considerable impact on the prevalence estimate obtained. Simple definitions, commonly used in research, performed poorly compared with the Rome III criteria. Studies estimating population prevalence of constipation should use definitions based on the Rome criteria where possible.

## Introduction

Constipation is a common condition in the community which represents a significant burden for both individuals and health care systems [1]. For the individual, constipation is associated with pain and symptoms which negatively impact quality of life [2]. From the health care system perspective, considerable costs are associated with the diagnosis and treatment of constipation [3–7]. Since constipation is such a burden, it is important to know its prevalence but estimating the prevalence of constipation in the community can be challenging.

Three large systematic reviews including 50 epidemiological studies of community-dwelling adult populations have shown that the prevalence varies widely, with estimates for constipation ranging from 2 to 35% [8–10]. This wide range may be in part due to differences in populations, because of various factors such as age groups, culture, diet and environment, but it may also be due to differences in the way constipation was defined in each study [8]. Although the Rome criteria have been developed for use as a standard definition of chronic constipation, most epidemiological studies have used a variety of other definitions of constipation. The various definitions used have included self-reported constipation or questions based on one or some of the symptoms as detailed in the Rome criteria.

The Rome criteria comprise a set of clinical symptoms which are internationally recognised as the gold standard in the diagnosis of chronic constipation [11, 12]. These criteria were first developed in 1994 (Rome I) and subsequently revised in 2000 (Rome II), 2006 (Rome III) and 2016 (Rome IV) [13]. Whilst useful in clinical research and pharmaceutical trials, the Rome criteria have been found cumbersome to use in clinical practice [13] and have not always been used in epidemiological research [14]. The majority of epidemiological studies have not investigated chronic constipation and have instead used simple definitions of constipation such as self-reported constipation over a defined time period, or specific symptoms, such as bowel motion frequency [8, 15].

It is not known to what extent the definition of constipation impacts prevalence estimates and whether any of these simple measures are valid alternatives to the Rome criteria for estimating the prevalence of chronic constipation. The aims of this study were to explore the impact that different definitions used to identify individuals with constipation have on population prevalence estimates and to compare the performance of simple definitions with the Rome III criteria.

## Methods

This study compared the prevalence of constipation in the study population using five simple definitions with the prevalence estimated using the Rome III criteria. The sensitivity, specificity and predictive values for each of the simple definitions were determined using the Rome III criteria as the gold standard for chronic constipation and modified Rome III criteria for sub-chronic constipation.

### Study population

Community-dwelling adults (aged 18 years and over) who were registered with a market research company (Research Now) in April 2015 were invited to participate in an online questionnaire exploring constipation and laxative use. The market research company received payment for the work and ensured that the final sample was representative of the Australian population with respect to gender, age and location by state. Informed consent from all participants was obtained prior to completion of the online questionnaire and participants were paid a nominal fee by the market research company as compensation for their time. Survey responses were confidential and the identity of the participants was not revealed to the researchers.

### Constipation definitions

#### Rome criteria

At the time that this survey was conducted (2015), the current Rome criteria were Rome III (Table 1). Chronic constipation was defined as meeting the Rome III criteria i.e. two or more symptoms as outlined in Table 1 for the last 3 months, with onset of symptoms being at least 6 months prior. A modification to the Rome III criteria was recently proposed for the identification of sub-chronic constipation whereby symptoms are assessed over 3 months as per the definition of chronic constipation but without the requirement for 6 months onset of symptoms [16]. This definition of sub-chronic constipation was also used since some simple definitions are based on a time period of 3 months. Both of these definitions were used as the gold standards in this study i.e. Rome III criteria for chronic constipation and the modified Rome III criteria for sub-chronic constipation.

**Table 1:** The Rome III diagnostic criteria for defining chronic constipation

Rome III Diagnostic Criteria [11]	
Diagnostic criteria^a^ 1. Must include *two or more* of the following: a. Straining during at least 25% of defaecations b. Lumpy or hard stools in at least 25% of defaecations c. Sensation of incomplete evacuation for at least 25% of defaecations d. Sensation of anorectal obstruction/blockage for at least 25% of defaecations e. Manual manoeuvres to facilitate at least 25% of defaecations (e.g. digital evacuation, support of the pelvic floor) f. Fewer than 3 defaecations per week. 2. Loose stools are rarely present without the use of laxatives 3. Insufficient criteria for irritable bowel syndrome	

^a^Criteria fulfilled for the last 3 months with symptom onset at least 6 months prior to diagnosis

In the online survey, validated questions regarding each of the symptoms specified in the Rome III criteria were used to determine chronic and sub-chronic constipation [11].

### Simple definitions

A literature search was conducted to identify simple definitions other than the Rome criteria, which have been used in studies reporting the prevalence of constipation. Five simple definitions were identified: self-reported constipation over the past 2 weeks, self-reported constipation over the past 3 months, self-reported constipation over the past 12 months, fewer than 3 bowel motions per week over the past 3 months and fewer than 3 bowel motions per week over the past 12 months.

In the online survey, self-reported constipation over 2 weeks, 3 months or 12 months was assessed using the questions: *“Have you felt constipated at any time during the last 2 weeks (or 3 months or 12 months)?”*. Constipation defined by fewer than 3 bowel motions per week over 3 months was assessed using the question: “*Over the last 3 months, do you often have fewer than 3 bowel movements each week?”.* Constipation defined by fewer than 3 bowel motions per week over 12 months was assessed using the question: *“In the last 12 months, how many bowel movements did you usually have each week?”*

### Sample size

Based on an estimated prevalence of 30% [17], a minimum of 2000 participants were required to give a prevalence estimate within 2 percentage points using a 95% confidence interval (Epi-Info Version 7, Centers for Disease Control & Prevention).

### Analysis

Descriptive statistics were used to describe the study population. Chi-squared testing was used to check the representativity of the study population against the national population in terms of gender, age, and location.

The prevalence of constipation in the study population was calculated for each definition as the number of individuals identified with constipation according to each definition, divided by the number of individuals in the study population.

To determine how well each definition performed at identifying constipation compared with the gold standards, the sensitivity, specificity, positive predictive value (PPV) and negative predictive value (NPV) were calculated for each simple definition using the Rome III criteria (chronic constipation) and modified Rome III criteria (sub-chronic constipation) as the gold standards. Sensitivity was calculated as the proportion of participants who were considered “constipated” by the simple definition out of those considered “constipated” by the gold standard. Specificity was determined as the proportion of participants who were considered “not constipated” according to the test definition out of those considered “not constipated” by the gold standard. The positive predictive value (PPV) was calculated as the proportion of participants considered “constipated” by the gold standard out of those considered “constipated” by the simple definition and the negative predictive value (NPV), the proportion of participants considered “not constipated” by the gold standard out of those participants considered “not constipated” by the simple definition [18].

A simple definition that could substitute for the Rome III criteria would have high values for sensitivity, specificity, PPV and NPV. Ideally each value should be close to 100% indicating that the definition is correctly identifying those with and without constipation according to the gold standard [19].

All statistical analysis was conducted using IBM SPSS Statistics (Version 22, IBM Corporation).

## Results

### Study population

The market research company invited 29,174 community-dwelling adults to participate in the online survey and the questionnaire was completed by 2376 (8.1%) respondents. After elimination of incorrectly completed questionnaires, the study population comprised 2024 participants. Participants were representative of the Australian community-dwelling adult population in terms of gender, age and location (Table 2). Half of the study population were aged over 45 years (50.6%) and half (50.7%) were female. In the past year, 13% of the study population had consulted a health care professional regarding constipation and 37% had used one or more laxatives.

**Table 2:** Study population characteristics (*n* = 2024)

Characteristic		% (Number of participants) *n* = 2024	National population [33]	*p* value
Gender:	Female	50.7% (1027)	50.6%	1
Age (years):				0.999
	18–24	12.4% (250)	12.4%	
	25–34	17.8% (361)	18.2%	
	35–44	17.6% (357)	18.8%	
	45–54	18.5% (374)	18.1%	
	55–64	14.6% (296)	15.3%	
	65+	19.1% (386)	17.2%	
Location (state):				0.999
	NSW	30.9% (627)	32.0%	
	Vic	24.9% (504)	24.9%	
	Qld	20.9% (423)	20.1%	
	SA	8.1% (163)	7.2%	
	WA	10.0% (203)	11.0%	
	Tas	2.1% (42)	2.2%	
	NT	0.9% (19)	1.0%	
	ACT	2.1% (43)	1.6%	
Self-rated health status:	Poor	4.0% (80)		
Comorbidities:	IBS^a^	9.3% (189)		
	Diabetes	7.5% (152)		
	Depression	12.9% (262)		
HCP^b^ consulted for constipation^c^		13.2% (268)		
Laxative use^c^		36.9% (747)		

^a^*IBS* Irritable bowel syndrome (medically diagnosed) ^b^*HCP* Health care professional ^c^In last 12 months

### Prevalence

Using the Rome III criteria, 24.0% of the study population had chronic constipation and 39.6% had sub-chronic constipation (Table 3). Using the simple definitions, the prevalence estimates varied six-fold, from 9.4% using “*fewer than 3 bowel motions per week in the past 12 months*” to 58.9% with “*self-reported constipation in the past 12 months*”. The prevalence estimate using “*self-reported constipation in the past 2 weeks*” (24.9%, 95% CI 22.9–26.8) was comparable to that that obtained by the Rome III criteria for chronic constipation (24.0%, 95% CI 22.1–25.9).

**Table 3:** Impact of constipation definition on prevalence estimate

Definition	% Prevalence estimate (95% Confidence interval)
Chronic constipation (Rome III criteria)	24.0 (22.1, 25.9)
Sub-chronic constipation (modified Rome III criteria)	39.6 (37.5, 41.7)
Self-reported constipation in the past 2 weeks	24.9 (22.9, 26.8)
Self-reported constipation in the past 3 months	29.2 (27.2, 31.2)
Self-reported constipation in the past 12 months	58.9 (56.8, 61.0)
Fewer than 3 bowel motions per week in the past 3 months	19.6 (17.9, 21.3)
Fewer than 3 bowel motions per week in the past 12 months	9.4 (8.1, 10.7)

The most common Rome III criteria symptoms reported by participants were straining, hard stools and a feeling of incomplete evacuation of stools, each of which were reported by approximately 80% of those with chronic constipation (Table 4). The least common symptom was the need for manual manoeuvres to assist defaecation. Approximately 40% of participants with chronic or sub-chronic constipation reported having fewer than 3 bowel motions per week.

**Table 4:** Prevalence of symptoms associated with constipation as per the Rome III criteria

Symptom	All participants (n = 2024)	Participants with chronic constipation according to the Rome III criteria (*n* = 485)	Participants with sub-chronic constipation according to the modified Rome III criteria (*n* = 801)
Fewer than 3 bowel motions per week	19.6% (397)	46.2% (224)	42.1% (337)
Straining	29.2% (590)	79.2% (384)	70.8% (567)
Hard stools	36.8% (744)	78.8% (382)	75.8% (607)
Incomplete evacuation	38.4% (778)	82.9% (402)	77.3% (619)
Perceived blockage	22.5% (457)	64.1% (311)	55.8% (447)
Manual manoeuvres	11.0% (222)	35.5% (172)	26.2% (210)

### Performance of simple definitions

No simple definition met our criteria for substitution of the Rome III criteria for either chronic or sub-chronic constipation i.e. none of the definitions had high enough values for sensitivity, specificity, PPV and NPV (Table 5).

**Table 5:** Sensitivity, specificity, positive and negative predictive values for the five simple definitions compared to the Rome III criteria and modified Rome III criteria as gold standards

Gold standard	Simple definition	Sensitivity (95% Confidence interval)	Specificity (95% Confidence interval)	Positive Predictive Value (PPV) (95% Confidence interval)	Negative Predictive Value (NPV) (95% Confidence interval)
Chronic constipation (Rome III criteria)	Self-reported constipation over past 2 weeks	64.5% (60.2, 68.7)	87.7% (85.9, 89.2)	62.2% (57.9, 66.4)	88.7% (87.0, 90.2)
	Self-reported constipation over past 3 months	72.0% (67.8, 75.8)	84.3% (82.4, 86.1)	59.2% (55.1, 63.0)	90.5% (88.9, 91.9)
	Self-reported constipation over past 12 months	91.1% (88.8, 93.4)	51.3% (48.8, 53.8)	37.1% (34.4, 39.9)	94.5% (93.1, 96.1)
	Fewer than 3 bowel motions per week over past 3 months	46.2% (41.8, 50.6)	88.8% (87.1, 90.2)	56.4% (51.5, 61.2)	84.0% (82.1, 85.7)
	Fewer than 3 bowel motions per week over past 12 months	17.3% (14.2, 20.1)	93.0% (91.7, 94.2)	44.0% (37.1, 51.1)	78.1% (76.2, 80.0)
Sub-chronic constipation (modified Rome III criteria)	Self-reported constipation over past 2 weeks	50.6% (47.1, 54.0)	92.0% (90.3, 93.4)	80.5% (76.8, 83.7)	39.6% (37.5, 41.7)
	Self-reported constipation over past 3 months	61.3% (57.9, 64.6)	91.9% (90.3, 93.3)	83.2% (80.0, 86.0)	78.4% (76.2, 80.4)
	Self-reported constipation over past 12 months	84.3% (81.6, 86.6)	57.7% (54.9, 60.5)	56.6% (53.8, 59.4)	84.9% (82.3, 87.1)
	Fewer than 3 bowel motions per week over past 3 months	42.1% (38.7, 45.5)	95.1% (93.7, 96.2)	84.9% (81.0, 88.1)	71.5% (69.2, 73.6)
	Fewer than 3 bowel motions per week over past 12 months	16.9% (14.4, 19.6)	95.4% (94.1, 96.5)	70.7% (63.9, 76.7)	63.7% (61.4, 65.8)

The simple definition with the highest sensitivity for identifying both chronic and sub-chronic constipation was “*self-reported constipation over past 12 months”*. This definition identified 91% of individuals who were considered to have chronic constipation according to the Rome III criteria. While the sensitivity of this definition was high, the specificity was much lower (51%) indicating that the definition incorrectly classified 49% of participants who were not constipated according to the Rome III criteria as being constipated. The PPV for this definition was 37%, finding that of all the participants considered constipated by this definition, only 37% were also considered constipated by the gold standard (Rome III). Similarly, for sub-chronic constipation, this definition correctly identified 84% of individuals but it had the lowest specificity (58%) and lowest PPV (57%) of any of the simple definitions when tested against the modified Rome III criteria.

The two simple definitions based on fewer than three bowel motions per week had high specificities, i.e. they correctly identified most participants who were not constipated using both the Rome III and modified Rome III criteria. However, the sensitivities associated with these definitions were very low (17 to 46%), showing that these definitions were unable to identify all of those constipated according to the Rome III criteria.

## Discussion

This study found that the definition of constipation used to estimate prevalence has considerable impact on the estimates of prevalence obtained. We found six-fold differences in the prevalence of constipation estimated using different definitions. Using the Rome III criteria in a large nationally representative sample of community-dwelling adults, we estimate that one in four adults is chronically constipated. None of the simple definitions tested in this study performed well enough in terms of sensitivity, specificity, PPV and NPV to be considered as suitable proxies for definitions of chronic or sub-chronic constipation based on the Rome III criteria.

Using the Rome III criteria, we estimated the prevalence of chronic constipation in the Australian community-dwelling adult population to be 24.0%. Previous studies have estimated the prevalence of chronic constipation among Australian adults to range from 2.8 to 30.7% [16, 17, 20–23]. Different definitions used in these studies may be the most important factor contributing to the wide range, however, differences in sample populations, sample sizes and data collection methods may also be relevant. Our survey was conducted online with a large nationally representative sample, whereas all previous Australian studies were mail surveys focussed on participants from certain geographical regions within Australia which were unlikely to be representative of the national population.

Although it is frequently difficult to achieve both high sensitivity and high specificity when testing against a gold standard [18], none of the commonly used simple definitions tested in our study adequately identified individuals with constipation compared with the Rome III criteria for either chronic or sub-chronic constipation. To some extent this might have been expected since we compared very simple definitions with a more complex definition as the gold standard but use of sensitivity, specificity, positive and negative predictive values is the appropriate method to quantify the accuracy of alternative diagnostic tests against a definitive gold standard diagnostic test [18]. We observed considerable variation in prevalence estimates with the different constipation definitions which indicates that differences in the way constipation is defined may explain the wide variation in the prevalence of constipation reported in the literature [8–10]. It also illustrates the importance of using a suitable definition to identify individuals with constipation when conducting prevalence studies. The diversity of estimates of constipation prevalence in the Australian community further illustrates the issue of different results obtained in different studies with different definitions of constipation.

When considering the prevalence of individual symptoms, the most common symptoms were straining, hard stools and incomplete evacuation which have similarly been reported as the three most common core symptoms of constipation in other studies [24]. Only 42 to 46% of participants who were regarded as constipated (sub-chronic or chronic) by the Rome III criteria reported experiencing fewer than 3 bowel motions per week. Also, less than 20% of all participants reported fewer than 3 bowel motions per week yet almost 60% had self-reported constipation in the last 12 months. Similar findings have been reported in Japan where 28% of participants in an online survey considered themselves to be constipated, but only 8% reported a bowel motion frequency of fewer than 3 per week [25]. In our study, simple definitions for identifying constipation which used bowel motion frequency performed poorly against the Rome III criteria, demonstrating that bowel motion frequency should not be used to identify constipation, unless used as one of the symptoms of the Rome criteria.

Other research has found that estimates of prevalence using self-reported constipation are greater than prevalence figures for chronic constipation derived from Rome criteria in the same population [26–29]. We found that self-reported constipation over 2 weeks approximated the Rome III prevalence estimate for chronic constipation. However, importantly both the sensitivity and PPV of the simple definition, “*self-reported constipation in the past 2 weeks”*, were low, indicating that this simple definition did not identify the same individuals with constipation as identified by the Rome III criteria. One factor to consider is that self-reported constipation is any constipation experienced during the defined time period whereas unmodified Rome criteria provide an indication of chronic constipation only. A further consideration is that with self-reported constipation, constipation is self-defined and consequently different individuals may have different perceptions of constipation [8, 30, 31]. It could be argued that this is the true definition of constipation which should be used in clinical practice but in prevalence studies of any constipation, our results suggest that defining constipation as *“self-reported constipation for the last 3 months”* might be considered as an alternative to other constipation definitions since it compared more favourably to the gold standards for both chronic and sub-chronic constipation than the other definitions in terms of sensitivity, specificity, PPV and NPV.

Our study showed that the period of time used in the simple definitions affected the estimated prevalence. Looking at the three definitions using self-reported constipation, as the time period increased the estimated prevalence also increased, as might be expected in any estimate of period prevalence [32]. Also, the time periods which have been used with self-reported constipation often differ to the periods specified in the Rome criteria. Many international epidemiological studies have used self-reported constipation over 12 months as the definition of any constipation but our results indicate that this may over-estimate the prevalence of constipation.

A major strength of our study is that it was conducted in a large population-based sample which was nationally representative in terms of age, gender and location of the Australian community-dwelling adult population. One limitation of our study was that although we asked if participants had been medically diagnosed with irritable bowel syndrome (IBS), we did not include questions regarding the Rome III diagnostic criteria for IBS. Consequently, our estimated prevalence of 24.0% using the Rome III criteria may include both functional constipation and constipation due to IBS. This may be one reason why our prevalence estimates for chronic and sub-chronic constipation were much higher than the most recent study in Australia where it was estimated that almost 4% of the population may experience constipation-predominant IBS [16]. Furthermore, it should be noted that, although our survey was conducted prior to publication of Rome IV criteria, the Rome IV criteria for functional constipation are essentially the same as Rome III criteria. [14]*.* A further limitation is the possibility that those with constipation were more likely to complete the questionnaire resulting in some selection bias which may in turn over-estimate the prevalence.

## Conclusion

This study highlights the importance of careful selection of the definition since it has a major bearing on the estimated prevalence of constipation. All of the simple definitions included in this study are commonly used in research, yet none provided a valid alternative to the Rome III criteria which are considered to be the gold standard for diagnosis of chronic constipation, or to the modified Rome III criteria for diagnosis of sub-chronic constipation.

## Abbreviations

IBS: Irritable bowel syndrome
NPV: Negative predictive value
PPV: Positive predictive value
